# P-1207. In Vitro Activity of Zoliflodacin against Neisseria gonorrhoeae Isolates Collected in 2022 from The United States

**DOI:** 10.1093/ofid/ofaf695.1400

**Published:** 2026-01-11

**Authors:** Sarah McLeod, Carmen Au, Meredith Hackel, Alison Luckey

**Affiliations:** Innoviva Specialty Therapeutics, Inc., Waltham, MA; Global Antibiotic Research and Development Partnership, Geneva, Geneve, Switzerland; IHMA, Schaumburg, IL; Global Antibiotic R&D Partnership (GARDP), Geneva, Geneve, Switzerland

## Abstract

**Background:**

Zoliflodacin is an oral, single-dose antibiotic being developed for patients with uncomplicated gonorrhea, including those infected with multidrug-resistant strains. Zoliflodacin is a first-in-class spiropyrimidinetrione that inhibits bacterial DNA biosynthesis primarily through the GyrB subunit of DNA gyrase. In this study, the in vitro activity of zoliflodacin against *Neisseria gonorrhoeae* collected in the US during 2022 was measured to verify continued activity against contemporary clinical isolates.

**Methods:**

A total of 123 *N. gonorrhoeae* isolates, provided by the US Centers for Disease Control and Prevention (CDC) as part of the CDC Gonococcal Isolate Surveillance Program (GISP), were tested for in vitro susceptibility to zoliflodacin and 8 comparators. Isolates originated from both males (79.7%) and females (20.3%) and mainly from urogenital body sites (92.7%). Minimum inhibitory concentrations (MICs) were determined using agar dilution according to CLSI guidelines (M07, 11^th^ ed.).

**Results:**

The zoliflodacin MIC_50/90_ values were 0.12/0.12 µg/mL (MIC range ≤0.008 – 0.25 µg/mL). Susceptibility to ceftriaxone and azithromycin was high at 100% and 97.6%, respectively. Conversely, 10% and 64% of isolates were susceptible to tetracycline and ciprofloxacin, respectively. Zoliflodacin maintained activity, with MIC_90_ of 0.12 µg/mL, against all 43 ciprofloxacin-resistant isolates and all 110 tetracycline-non-susceptible isolates, and an MIC range of 0.12 – 0.25 µg/mL against all 3 azithromycin-non-susceptible isolates. There was no difference in the zoliflodacin MIC_90_ for isolates from males and females.

**Conclusion:**

For *N. gonorrhoeae* clinical isolates collected in the US during 2022, the zoliflodacin MIC_50/90_ and MIC range were consistent with the potency observed during 2020 and 2021 US surveillance studies and demonstrate activity against antibiotic-resistant isolates and clinical isolates from males and females. These data support the continued development of zoliflodacin for patients with uncomplicated gonorrhea.

**Disclosures:**

Sarah McLeod, PhD, Innoviva Specialty Therpeutics: Employee|Innoviva Specialty Therpeutics: Stocks/Bonds (Public Company)

